# NURSE-LED INTERVENTIONS FOR ADULTS WITH POSTSTROKE COGNITIVE IMPAIRMENT: A SCOPING REVIEW

**DOI:** 10.1093/geroni/igae098.2955

**Published:** 2024-12-31

**Authors:** Azza Alharrasi, Marie Boltz

**Affiliations:** The Pennsylvania State University, University Park, Pennsylvania, United States; The Pennsylvania State University, University Park, Pennsylvania, United States

## Abstract

**Background:**

Stroke is one of the leading causes of death and disability worldwide. Post-stroke cognitive impairment (PSCI) is a common complication of stroke, associated with increased cost of care, hospitalizations, and care dependency. Nurses play a central role in rehabilitation of adult’s post-stroke; however, is unknown about nursing-led interventions in promoting global cognition. The purpose of this review to explore Nurse-led interventions (NLI) and explore their impact up on global cognition and discrete cognitive domains.

**Methods:**

The search strategy identified studies within PubMed, CINHAL, and Web of Science. The search was conducted on October 3rd, 2023, and November 7th, 2023, and 12 articles were identified. Inclusion criteria were the studies that delivered nursing interventions among adults with post-stroke cognitive impairment with the outcome of improving cognitions or discrete cognitive domains. Data extraction of illegible studies that met the inclusion criteria guided by the interventions, duration and follow-up, global cognition, discrete cognitive domains.

**Results:**

The results demonstrated the significant improvement in the global cognition, discrete cognitive domains, activity of daily livings (ADLs), motor function, and quality of life. Results underscore the need for early cognitive screening and rehabilitation. The results found that there is a gap in knowledge regarding the long-term benefits or effectiveness of NLI upon PSCI.

**Conclusion:**

The review suggests that more rigorous multi-center research studies need to be conducted on the NLI for adults with PSCI that should be with adequate sample size.

